# Supplementary material for: MetaRibo-Seq measures translation in microbiomes
Source: Nat Commun. 2020 Jun 29;11:3268. doi: 10.1038/s41467-020-17081-z (PMC7324362; doi:10.1038/s41467-020-17081-z)
Supplement: Supplementary file 10 — Supplementary Data 7 [file 41467_2020_17081_MOESM10_ESM.zip › File2/Confidence_VeryHigh_Taxonomy/321930_out.krona.html]

Javascript must be enabled to view this page.

members
magnitude
magnitudeUnassigned
count
unassigned
taxon
rank

321930\_out

16

2
16
superkingdom

phylum
16
1239

class
186801
16

order
16
186802

16
186806
family


SRS014979\_contig\_number\_contig-100\_41415.41416SRS016954\_contig\_number\_contig-100\_6691.6691SRS050422\_contig\_number\_contig-100\_30478.60937SRS143991\_contig\_number\_contig-100\_17436.17436
genus
4
1730
16

165185
5
species

SRS019068\_contig\_number\_43346SRS1041091\_contig\_number\_385SRS143342\_contig\_number\_16603SRS148721\_contig\_number\_29420SRS149879\_contig\_number\_6978

species

SRS016495\_contig\_number\_contig-100\_5334.5334SRS018427\_contig\_number\_contig-100\_13377.13377SRS049712\_contig\_number\_contig-100\_11617.41900SRS053214\_contig\_number\_14261SRS077502\_contig\_number\_13671SRS144183\_contig\_number\_33469SRS148159\_contig\_number\_3054
39488
7
